# Screening for Alcohol Use Disorder Among Hospitalised Patients: Learning from a Retrospective Cohort Study in Secondary Care

**DOI:** 10.3390/jcm13247617

**Published:** 2024-12-13

**Authors:** Mohsan Subhani, Dipaka Rani Nath, Usman Talat, Aqsa Imtiaz, Amardeep Khanna, Awais Ali, Guruprasad P. Aithal, Stephen D. Ryder, Joanne R. Morling

**Affiliations:** 1 Nottingham Digestive Diseases Biomedical Research Centre (NDDC), School of Medicine, University of Nottingham, Nottingham NG7 2GT, UK; guru.aithal@nottingham.ac.uk (G.P.A.); stephen.ryder4@nhs.net (S.D.R.); joanne.morling@nottingham.ac.uk (J.R.M.); 2 NIHR Nottingham Biomedical Research Centre, Nottingham University Hospitals NHS Trust and the University of Nottingham, Nottingham NG7 2GT, UK; dipikanath087@gmail.com (D.R.N.); amardeep.khanna7@nhs.net (A.K.); awais.ali15@nhs.net (A.A.); 3 Dyson School of Design Engineering, Imperial College London, London SW72BX, UK; u.talat@imperial.ac.uk; 4 School of Medicine, Fatima Jinnah Medical University, Lahore 54000, Pakistan; aqsaimtiaz376@gmail.com; 5 Division of Epidemiology and Public Health, Nottingham University Hospitals NHS Trust and the University of Nottingham, Nottingham NG7 2RD, UK

**Keywords:** alcoholism, mental health, liver disease, secondary care, retrospective

## Abstract

**Background:** Excessive alcohol consumption is among the leading causes of hospitalisation in high-income countries and contributes to over 200 medical conditions. We aimed to determine the prevalence and characteristics of alcohol use disorder (AUD), describe the distribution of AUD in ICD-10 discharge diagnosis groups and ascertain any relationship between them in secondary care. **Methods:** The study group was a retrospective cohort of adult patients admitted to Nottingham University Hospital (NUH) between 4 April 2009 and 31 March 2020. Uni- and multivariable analysis was performed to determine the relationship between AUD and covariable high-risk characteristics and describe the distribution of AUD in ICD-10 discharge diagnosis groups defined by an alcohol-attributable fraction. **Results:** A total of 44,804 patients (66,440 admissions) were included, with a mean age of 63.1 years (SD ± 19.9); of these, 48.0% *(n* = 20,863) were male and 71.2% were (*n* = 30,994) white. AUDIT-C was completed in 97.1% (*n* = 43,514) of patients, and identified 16.5% (*n* = 7164) as having AUD, while 2.1% (*n* = 900) were found to be alcohol-dependent. In patients with AUD, 4.0% (*n* = 283) had an ICD-10 diagnosis that was alcohol-specific and 17.5% (*n* = 1255) were diagnosed with alcohol-related disorders; the remainder were not diagnosed with either disorder. Two-thirds (64.7%) of the patients with AUD had associated mental and behavioural disorders. Multivariable logistic regression analysis revealed that patients aged 60–69 had the highest risk of AUD (OR 4.19, 95% CI 3.53–4.99). Being single (OR 1.18, 95% CI 1.11–1.26) and a history of emergency admission (OR 1.21, 95% CI 1.14–1.29) were associated with increased odds of AUD. Conversely, females compared to males (OR 0.34, 95% CI 0.35–0.39), individuals from minority ethnic backgrounds compared to white Caucasians (OR 0.39, 95% CI 0.35–0.45), and those from more deprived areas (IMD quintile 1: OR 0.79, 95% CI 0.74–0.86) had lower odds of AUD. **Conclusions:** One in six admitted patients had AUD, with a higher risk in males, ages 60–69, and emergency admissions. Mental disorders are highly prevalent among hospitalised patients with AUD. The performance of the AUDIT-C score varied among hospitalised patients based on their ICD-10 diagnosis, which should be considered when implementing universal alcohol screening in these settings.

## 1. Introduction

Excessive alcohol consumption is among the most common reasons for hospitalisation. In 2019, 7.4% of all hospital admissions in England were related to alcohol [1]. Alcohol-specific mortality is now at an all-time high since 2001, with a 20% increase observed in 2020 compared to 2019 (13.0 versus 11.0 per 100,000) [2]. This has huge cost implications for the UK’s National Health Service (NHS). It is estimated that 1.3 million alcohol-related hospital admissions occur annually, amounting to a cost for the NHS of GBP 3.5 billion each year [3,4]. According to the statistics provided by the World Health Organisation (WHO) for 2018, globally, alcohol-related disorders (ARDs) lead to >3 million deaths each year, contributing to 7% of premature deaths (age ≤ 65 years), and 132.6 million disability-adjusted life years (DALYs). The overall alcohol-related mortality is ahead of other common causes like diabetes, HIV, and tuberculosis [4].

Alcohol contributes to over 200 different medical conditions, of which at least 25 are wholly attributable to alcohol [5]. To manage these conditions, patients often require hospitalisation, which presents a distinct opportunity for effective intervention in alcohol use disorder (AUD) [6]. The brief interventions provided by healthcare workers are proven to reduce alcohol-related harm [7]. This puts healthcare staff in a unique position to intervene in alcohol-related disease processes, facilitate recovery, and prevent future harm [8]. However, AUD detection rates in both primary and secondary care environments are poor [9,10]. From the UK’s primary care electronic health record (EHR) data, it was observed that alcohol use was infrequently documented. In 2018, only 50% of 1.8 million patients had any record of information about their alcohol consumption within the preceding five years [9]. A National Confidential Enquiry into Patient Outcome and Death in 2013 highlighted the inadequacy in the screening and management of patients hospitalised for harmful alcohol intake [11].

This could be due to inadequate knowledge, awareness and negative attitudes toward patients with AUD [12]. Lack of patient and service provider engagement and failure of early identification is inevitable if there is no recognition of where and how these patients have interacted with healthcare services [13]. An in-depth understanding of AUD in hospital settings and associated high-risk patients and environment-related factors are critical for developing targeted alcohol services [14].

Early identification of high-risk drinking behaviours followed by interventions is pivotal in preventing future alcohol-related harm [15]. Universal alcohol screening of hospitalised patients, performed using validated instruments, demonstrates a significantly higher prevalence of AUD compared to conventional International Classification of Diseases (ICD-10) diagnosis codes [16]. The Alcohol Use Disorders Identification Test (AUDIT) is a ten-item questionnaire developed by the WHO to screen for hazardous, harmful, and dependent alcohol consumption [17]. The Alcohol Use Disorders Identification Test Consumption (AUDIT-C) is an abbreviated version of the AUDIT which consists of three consumption questions from the full AUDIT [18]. The National Institute for Health and Care Excellence (NICE) guidelines recommend that adults with a high level of alcohol intake should be screened for AUD and be offered intensive structured community-based interventions (with or without medical therapy), as these provide the best chance of achieving and maintaining abstinence from alcohol [19].

The use of AUDIT-C has been validated in primary care and research settings [15]. However, the performance of the AUDIT-C score compared to wholly alcohol-attributable and partly alcohol-attributable conditions in a secondary care cohort has not previously been demonstrated. The current study aims to address four research questions:

(i) What is the prevalence of and what are the characteristics of AUD among hospitalised patients?
(ii) How is AUD distributed among ICD-10 discharge diagnoses of alcohol-specific, alcohol-related, and non-alcohol-specific or alcohol-related admissions?
(iii) What high-risk shared characteristics are associated with AUD?
(iv) What are the acceptance rates of universal alcohol screening among these patients?

## 2. Methods

The study was conducted at Nottingham University Hospital (NUH), England, United Kingdom. NUH provides care to a population of approximately 700,000. Local institutional ethical approval was obtained (Registration Number: 20-728C). As the study analysed anonymised retrospective data from routine electronic health records, individual patient consent was not required. Strengthening The Reporting of Observational Studies in Epidemiology (STROBE) reporting guidelines were followed throughout the manuscript.

The retrospective cohort encompassed all adult patients aged 18 years and over admitted to NUH from 1 April 2019 to 31 March 2020. Universal alcohol screening (UAS) by AUDIT-C has been implemented at NUH since 2018. All patients have a mandatory electronic alcohol assessment by the admitting staff nurse. An AUDIT-C scores are defined as follows: 0–4 screen = negative for AUD; ≥5 screen = positive for AUD (increased risk score 5–7, high-risk score 8–10, alcohol-dependent score 11, 12) (Supplementary Table S1) [18].

All patients admitted to NUH during the 12-month study period who underwent alcohol assessment using the AUDIT-C score were included. As this was an observational study, no formal sample size calculation was conducted. The 12-month duration was selected to mitigate seasonal variation and ensure comprehensive annual data. The study dates were set pre-pandemic to avoid potential biases from pandemic-related effects.

At NUH, patients with an AUDIT-C score of 8 and above are referred to the hospital-based alcohol care team. The alcohol care team conduct a structured assessment of the patient and offer a variety of treatments such as psychological interventions, recovery support, and pharmacological intervention. The specific treatment options are based on individual patients’ needs. At discharge, any patients who require ongoing support are referred to community-based alcohol services.

Within this study, the term ‘alcohol use disorder (AUD)’ was used to represent and discuss the results of the AUDIT-C score and ‘alcohol-attributable disorders (AADs)’ encompassed broader alcohol-associated problems including alcohol-related liver disease (ARLD) and alcohol use disorder (AUD).

### 2.1. Data Source and Variables

Hospital-based activity and the access team facilitated the extraction of data from the electronic medical record (EMR). The EMR holds information on the patient’s discharge diagnosis, alcohol assessment, and demographics. Anonymised data on age, sex, ethnicity, civil status, mode of admission, AUDIT-C score at admission, discharge diagnosis (ICD-10 version 5), inpatient speciality of care, length of stay, number of hospital admissions, Lower Super Output Area (LSOA) and Indices of Multiple Deprivation (IMD) was extracted. The number of admissions was the total number of hospital inpatient visits a patient had during the study period.

As per Public Health England (PHE) guidance (2014 and 2020) [20], the diagnoses were defined from ICD-10 coding as alcohol-specific (wholly alcohol-attributable) where alcohol was the sole cause and their alcohol-attributable fraction was 1.0 (100 percent) (Supplementary Table S2), or alcohol-related (partly alcohol-attributable), where alcohol was contributory but not the sole cause (Supplementary Table S3) [20].

Lower Super Output Area (LSOA) is a metric created by the Office for National Statistics (ONS) to describe the statistics of a small area with an average of approximately 1500 residents or 650 households. As per the 2011 Census, there are 32,844 LSOAs in England, which were used to determine Indices of Multiple Deprivations (IMDs) 2015 [21]. Deprivation was assigned by using the IMD 2015 quintile, as provided by PHE, based on the LSOAs of residence. The IMD combines information from seven domains and produces an overall measure of deprivation. IMD ranks the scores to produce quintiles with 1 equal to the most deprived 20% and 5 equal to the least deprived 20% of neighbourhoods nationally.

### 2.2. Statistical Analysis

The primary outcomes of this study were (a) to describe the prevalence of alcohol use disorder (AUD) and characterise its features among hospitalised patients; and (b) to analyse the distribution of AUD among ICD-10 discharge diagnoses categorised as alcohol-specific, alcohol-related, and non-alcohol-specific or alcohol-related admissions.

The secondary outcomes included identifying shared high-risk characteristics commonly associated with AUD and evaluating the acceptance rates of universal alcohol screening, as assessed by the AUDIT-C questionnaire, among the patient population during the study period.

Quantitative variables that followed a normal distribution were summarised as the mean ± standard deviation (SD), while those with a non-normal distribution were presented as median ± interquartile range (IQR). The correlation between the AUDIT-C score and normally distributed quantitative variables was assessed using parametric tests, including Pearson’s correlation coefficient, an unpaired *t*-test, and ANOVA. For non-normally distributed variables, non-parametric tests including Spearman’s correlation coefficient and the Mann–Whitney U test were applied. Categorical variables were analysed using the Chi-Squared test, with results reported as absolute and relative frequencies, along with 95% confidence intervals. To assess the prevalence of AUD in various risk groups, patient characteristics were compared between those diagnosed with AUD based on the AUDIT-C score and those with ICD-10 discharge diagnoses of alcohol-specific, alcohol-related, and non-alcohol-specific or alcohol-related admissions.

Multivariable logistic regression analysis was performed to examine the relationship between AUD and patient characteristics. This analysis controlled for potential confounders, allowing for the identification of independent factors associated with AUD, while mutually adjusting for demographic, clinical, and diagnostic variables in the dataset. The lowest-risk group (no AUD) was used as a control.

The statistical analysis was conducted using IBM SPSS Statistics version 26 and GraphPad Prism Version 9.

## 3. Results

### 3.1. Description of the Cohort and Epidemiology of AUD

**Figure 1:**
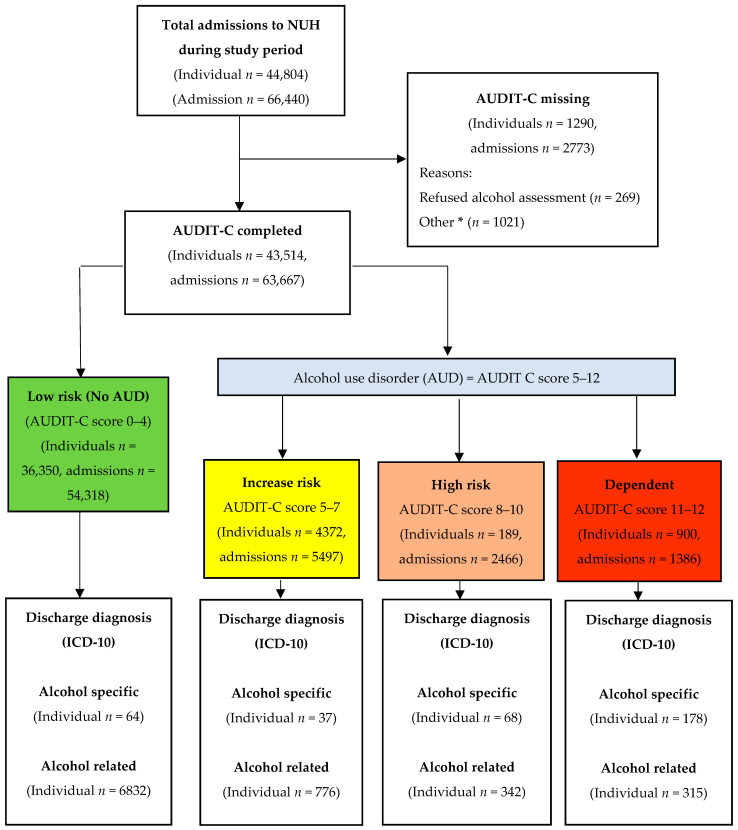
Participants flow diagram: Alcohol use disorder (AUD) was defined by an AUDIT-C score of 5–12 and split into three categories: increased risk, high-risk, and alcohol-dependent. An AUDIT score of 0–4 was deemed low risk for AUD. * Unconscious patients, critically unwell patients, and patients with cognitive impairment.

A total of 44,804 patients accounting for 66,440 admissions were included. Of these, 269 (0.60%) declined to complete the alcohol assessment, and 1021 (2.28%) did not complete the AUDIT-C due to other reasons. The analysis cohort therefore comprised alcohol assessment by AUDIT-C in 43,514 patients, comprising 63,667 admissions (Figure 1). The mean age of the cohort was 63.1 years (SD ± 19.9); 48.0% (*n* = 20,863) were male; 71.2% (*n* = 30,994) were white, and 44.7% (*n* = 17,819) were from two most deprived quintiles.

In the whole cohort, based on the AUDIT-C score, 16.5% (*n* = 7164) had AUD, of which 8.6% (*n* = 5497) were at increased risk, 3.9% (*n* = 2466) were classed as high-risk, and 2.2% (*n* = 1386) were alcohol-dependent. Among patients with AUD, 4.0% (*n* = 283) had alcohol-specific, 17.5% (*n* = 1255) alcohol-related, and 78.5% (*n* = 5626) had neither alcohol-specific nor alcohol-related discharge diagnoses. The baseline characteristic of the cohort is provided in Table 1 and for individual AUD risk groups (low-risk, increased risk, high-risk, and alcohol-dependent) in the Supplementary Materials Table S4.

#### 3.1.1. No AUD (Low-Risk) Versus AUD

The patients with AUD were significantly younger than those with no AUD (low risk), with a mean age difference of 8.6 years (SD ± 0.25, *p* < 0.001). Patients with AUD compared to the no AUD group were more likely to be male (*p* < 0.001), of white ethnicity (*p* < 0.001), not in a relationship (*p* < 0.001), cared for by medical specialists (*p* < 0.001), have a shorter length of stay (*p* < 0.001), and have mental and behavioural disorders (*p* < 0.001). The difference between deprivation quintiles and mode of admission was non-significant (Table 1).

#### 3.1.2. Individual AUD Risk Groups

Among individual AUD risk groups (low-risk, increased risk, high-risk, and alcohol-dependent), the alcohol-dependent group was the youngest (mean age 53.8 years ± 14.2), and had the highest proportions of males (73.1%) and patients of white ethnicity (93.6%). The alcohol-dependent patients compared to those with no AUD were from the most deprived neighbourhoods (IMDQ-1 38.3%) and were more likely to be not in a relationship (65.7%), admitted as a medical emergency (76.8%) and cared for by medical specialists (69.7%). In increased- and high-risk groups, surgery compared to medicine was the most common inpatient speciality of care (56.7% vs. 43.3% and 52.2% vs. 47.8%) (Supplementary Table S4).

#### 3.1.3. Inpatient Speciality of Care for Patients with AUD

**Figure 2:**
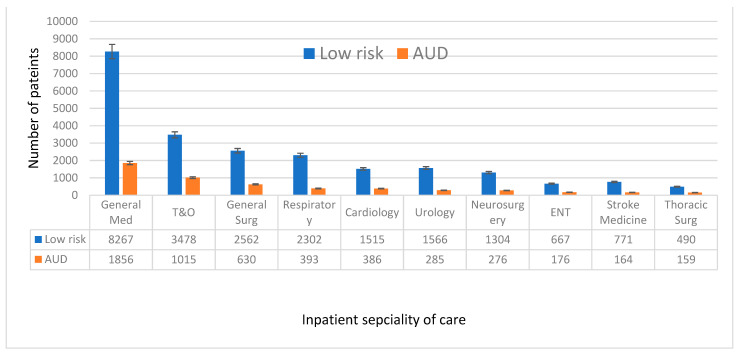
Top ten inpatient speciality of care: low risk versus alcohol use disorder (AUD).

The most common inpatient speciality of care for patients with AUD was general medicine (*n* = 1856, 27.2%), followed by trauma and orthopaedics (*n* = 1015, 14.9%), general surgery (*n* = 630, 9.2%), respiratory medicine (*n* = 393, 5.8%), and cardiology (*n* = 386, 5.6%). The top ten inpatient specialities of care for the AUD group compared to no AUD group are given in Figure 2 and for individual AUD risk groups in Supplementary Materials Figure S1.

### 3.2. AUD Amongst Discharge Code Type Based on the Alcohol-Attributable Fraction

#### 3.2.1. Alcohol-Specific (Wholly Alcohol-Attributable) Discharge Diagnosis

Among the whole cohort, 347 (0.8%) patients had a primary ICD-10 discharge diagnosis of alcohol-specific disorders, of which, based on AUDIT-C, 64 (18.4%) were low-risk and 283 (81.6%) had AUD. In patients with AUD, mental and behavioural disorders due to the use of alcohol were the most common alcohol-specific discharge diagnoses (*n* = 183, 64.7%).

Comparing those with AUD to the no-AUD group revealed that they were more likely to have mental and behavioural disorders due to the use of alcohol (64.7% vs. 39.1, *p* < 0.001) and gastrointestinal disorders caused by alcohol (11.7% vs. 7.8%, *p* < 0.001), whereas patients with liver disorders due to alcohol were more likely to be screened as a low risk than AUD (51.6% vs. 23.3%, *p* < 0.001) (Table 2).

#### 3.2.2. Alcohol-Related (Partly Alcohol-Attributable) Discharge Diagnosis

Among the whole cohort, 8087 (18.6%) had a primary ICD-10 discharge diagnosis of partly alcohol-related disorders, of which based on AUDIT-C, 6832 (84.5%) had no AUD, and 1255 (15.5%) had AUD (Table 3). In patients with AUD, cardiovascular diseases were the most common alcohol-related discharge diagnosis (*n* = 668, 53.2%), followed by respiratory infection (*n* = 240, 19.1%), malignant neoplasm (*n* = 174, 13.9%), and digestive diseases (*n* = 140, 11.2%).

#### 3.2.3. Non-Alcohol-Specific and Non-Alcohol-Related Discharge Diagnosis

Among the whole cohort, 35,080 (80.6%) patients had a primary ICD-10 discharge diagnosis which was neither alcohol-specific nor alcohol-related, of which, based on AUDIT-C, 29,454 (84.0%) patients were low-risk, and 5626 (16.0%) had AUD. In patients with AUD injury, poisoning and certain other consequences of external causes were the most common ICD-10 diagnoses (*n* = 1390, 24.7%), followed by diseases of musculoskeletal and connective tissue (*n* = 615, 10.9%), neoplasm (*n* = 591, 10.5%), diseases of the digestive system (*n* = 585, 10.4%), and abnormal clinical and laboratory findings (*n* = 562, 10.0%).

#### 3.2.4. Distribution of Alcohol-Specific Conditions Among the No AUD (Low-Risk) Group

Among patients who had no AUD, 64 (0.18%) had alcohol-specific ICD-10 discharge diagnoses. Alcohol-related liver disease was the most common alcohol-specific diagnosis (*n* = 33, 51.6%), followed by mental illnesses (*n* = 25, 39.1%). A detailed breakdown of the distribution of alcohol-specific conditions amongst individual risk groups is provided in Supplementary Materials Table S5.

### 3.3. Multivariable Logistic Regression Analysis

The results of multivariable logistic regression analysis (Table 4) showed that amongst all age groups, patients aged 60–69 years had the highest odds of having AUD (OR 4.19, 95% CI 3.53–4.99, *p* < 0.001). Females had OR 0.34 (95% CI 0.35–0.39, *p* < 0.001) lower odds of AUD compared to males. Minority ethnicity patients had OR 0.39 (95% CI 0.35–0.45, *p* < 0.001) lower odds of AUD compared to white patients. The odds of AUD decreased with increasing deprivation (*p* < 0.001). Other factors contributed to significantly higher odds of AUD, as follows: patients not in a relationship had an OR 1.18 (95% CI 1.11–1.26, *p* < 0.001) and emergency admissions OR 1.21 (95% CI 1.14–1.29, *p* < 0.001). Patients receiving specialist medical care had OR 0.85 (95% CI 0.80–0.90, *p* < 0.001), that indicated lower odds of AUD compared to those who underwent surgery. The multivariable logistic regression analysis for individual risk groups is provided in the Supplementary Material Table S6.

## 4. Discussion

This study demonstrates that one in six admitted patients (14.7%) were diagnosed with AUD based on AUDIT-C, which is significantly higher than the previously reported 7.4% of alcohol-related hospital admissions in the UK [1]. AUD was present in 81.6% of patients with alcohol-specific diagnoses and 15.5% of partly alcohol-related diagnoses. Alcohol assessment via the AUDIT-C was completed in over 97% of admitted patients, with less than 1% refusing to complete the AUDIT-C questionnaire. Acceptance rates were similar to previously reported community studies [15]. Universal alcohol screening provides a unique opportunity for early detection of AUD followed by NICE-recommended intensive structured interventions (with or without medical therapy), as these provide the best chance of achieving and maintaining abstinence from alcohol [19].

Almost two-thirds of patients with AUD had concomitant mental and behavioural disorders. This finding emphasises the importance of commissioning targeted services for mental health and alcohol support to at-risk populations to stem the tide of multi-morbidity, as highlighted by a recent report from The Royal College of Psychiatry UK [22]. Previous epidemiological studies have demonstrated a high prevalence of psychiatric disorders in patients with AUD and subsequent poorer outcomes [23]. The lifetime prevalence of AUD for patients with major depressive disorders ranges from 27 to 40%; in patients with anxiety disorders, its prevalence ranges from 20 to 40%, and in patients with post-traumatic stress disorders, its prevalence ranges from 34 to 55% [23]. This demonstrates the importance of integrated treatment strategies addressing both AUD and mental health, which have been reported to improve outcomes [24,25].

Alcohol use disorder (AUD) was identified in all groups of patients irrespective of their ICD-10 discharge diagnosis. In the alcohol-specific group, the majority of patients (81.6%) had AUD, and, amongst those who had no AUD (18.4%), ARLD was the leading diagnosis. One possible explanation for this could be that these patients had a history of AUD and subsequently had a diagnosis of ARLD—the diagnosis of ARLD may have led them to either stop drinking or change their drinking behaviour [26,27]. This may explain why they were classed as low-risk according to the AUDIT-C and were given wholly alcohol-attributable diagnoses. This also raises the possibility of missed opportunities; a recent study showed that patients who died of ARLD had multiple interactions with healthcare professionals prior to their death. Over half of them were only diagnosed with ARLD in the six months prior to their death [28]. If a universal alcohol screening policy had been adopted, these patients may have been identified earlier, at a stage where medical and behavioural intervention could have made a meaningful difference to the outcome [3]. Additionally, AUDIT-C relies on self-reported alcohol intake, which can be affected by recall and social desirability biases, leading to underestimation, particularly in a context where stigma still exists [29]. Studies highlight the tool’s limitations when applied to subgroups like pregnant individuals or the elderly—populations for whom AUDIT-C was not originally designed—emphasising the need for tailored approaches [30,31].

It is important to highlight that a significant proportion of patients who had an ICD-10 discharge diagnosis of alcohol-related disorders (15.5%) and non-alcohol specific or non-alcohol-related disorders (16.0%) had concomitant AUD. Although the proportion of patients with AUD in these groups was smaller, in the context of absolute numbers, these were much larger cohorts. The overarching worry is that if we restrict patient identification to ICD-10 diagnostic codes, this may lead to a significant proportion of patients with AUD being missed; alternatively, patients who are at low risk of harmful alcohol intake may be erroneously identified and end up receiving unwarranted interventions. These findings suggest that healthcare professionals should maintain a high index of suspicion for AUD among hospitalised patients.

Over half (52.0%) of the admitted patients were female, but the majority (67.7%) of patients who had AUD were male, of white ethnicity and in their 50s. The gender, ethnicity and age distribution were consistent with previously reported evidence [32,33]. Among the top ten inpatient specialities caring for AUD-positive patients, six were surgical. General medicine was most common (*n* = 1856, 27.2%), followed by trauma and orthopaedics (*n* = 1015, 14.9%), and then general surgery (*n* = 630, 9.2%).

The multivariable logistic regression analysis identified several key factors associated with AUD among hospitalised patients. Younger age, male sex, and being in a relationship were associated with higher odds of AUD, while patients from minority ethnicity backgrounds had lower odds of AUD compared to white patients. Emergency admissions and longer hospital stays were also significant predictors of AUD. These findings suggest that demographic, social, and clinical factors play crucial roles in identifying patients at risk of AUD in secondary care settings [34]. Interestingly, in this cohort, increased deprivation was associated with reduced odds of alcohol use disorder (AUD), with individuals from the most deprived neighbourhoods showing the lowest risk. However, patients with alcohol dependence were predominantly from the most deprived areas and exhibited a higher prevalence of co-existing mental health disorders. A survey conducted by the WHO in 2022 demonstrated that higher socioeconomic status was associated with a higher prevalence of excessive alcohol consumption, while individuals from less affluent socioeconomic backgrounds were more likely to engage in heavy episodic drinking [35].

### 4.1. Strengths and Limitations

This study contributes to an under-researched area by investigating the AUDIT-C tool in the largest cohort to date. This cohort encompassed 44,804 hospitalised adults with 66,440 admissions over a one-year period for alcohol-specific and alcohol-related conditions. With a high completion rate (>97%) among patients aged 18 years and older, AUDIT-C demonstrated effectiveness in identifying risky and problematic alcohol consumption, including cases that might otherwise remain undetected. AUDIT-C offers significant clinical value by facilitating early intervention and tailored care, particularly for patients with concurrent mental health conditions, improving overall outcomes and enhancing alcohol-related healthcare services in hospital settings.

The primary limitation of this study is its retrospective design, which poses risks such as information and selection biases [36]. While blinding the data extractor to study outcomes mitigated some bias, retrospective studies lack control over the accuracy and consistency of data collection, which may lead to missing or incomplete records. Additionally, the study relied on routinely collected clinical data, which may not capture nuanced patient behaviours or unrecorded variables influencing AUD. Although the validated AUDIT-C tool minimised misclassification, retrospective analyses are prone to recall bias, particularly with regard to self-reported alcohol consumption data [31]. Lastly, retrospective studies do not allow for direct causal inferences, limiting the ability to establish definitive relationships between AUD and associated factors.

The single-centre nature of the study and its predominantly white population may limit the generalisability of the results. Although 43% of Nottingham City’s population identified as belonging to ethnic minority groups in the 2021 National Census, only 10% of the study population represented minority ethnicities, indicating potential underrepresentation in this cohort [37]. Nottingham is the 11th most deprived area in England, with 30% of neighbourhoods among the country’s most disadvantaged 10% [38]. These factors emphasise the need for caution when extrapolating results to other populations and settings.

Finally, the ICD-10-based discharge diagnosis code has a risk of misclassification; however, several studies have demonstrated the high positive predictive value of alcohol-specific and alcohol-related codes [39]. This means we are confident the results are truly representative of patients with alcohol-attributable disorders. While the study effectively highlights alcohol use disorder distribution among hospitalised patients, it lacks information on the specific alcohol interventions provided and does not track the long-term impact of screening on patient outcomes.

### 4.2. Implications

The study proposes a pathway for alcohol screening among hospitalised patients. AUD was present across all groups of patients, irrespective of their discharge diagnosis. This is important when implementing policies on the provision of related services in hospital settings, such as NHS England’s latest initiative to screen hospitalised alcohol-dependent patients for liver fibrosis [40].

Alcohol contributes to a variety of medical conditions and managing some of these patients often requires hospitalisation [5]. Many of these conditions, such as ARLD, are diagnosed at a late stage [28]. Hospitalisation is often a “teachable moment” and can be used as a catalyst to change drinking behaviours [41]. This puts healthcare staff in a unique position to intervene in the alcohol-related disease process and prevent future harm [8]. This study presents a strong case that universal alcohol screening of hospitalised patients should be adopted nationwide, as it enhances the pickup rate of AUD and creates a window of opportunity to intervene.

The message that health improvement is the responsibility of all healthcare professionals is not yet fully embedded, despite assurances to the contrary. NICE recommends that every healthcare professional providing care to these patients should be competent at screening for harmful alcohol use and delivering brief advice [42]. Moreover, the 2010 position statement from The Royal College of Surgeons of England stressed the surgeon’s role in capitalising on “teachable moments” by screening patients for harmful alcohol intake, followed by a brief intervention [43]. Adopting a structured alcohol screening programme significantly improved trauma patient alcohol screening rates by 86% compared to prior methods [44]. In order to address alcohol-related conditions, treatment services must be more accessible, and education for healthcare professionals explaining how to integrate early diagnosis of AUD and interventions into their practice should be mandatory [45].

The use of AIBA has a proven role in reducing alcohol consumption and subsequent harm [7]. To address harmful alcohol intake effectively, the hospital-based alcohol care team needs to be more accessible. Healthcare professionals across all disciplines need to be educated on AIBA [45]. There is a growing body of evidence supporting clinician-integrated multidisciplinary care models to provide person-centred care for alcohol use disorder [46,47].

## 5. Conclusions

This study highlights the high prevalence of AUD among hospitalised patients, with one in six admitted patients being diagnosed with AUD using the AUDIT-C tool. AUD was most common in alcohol-specific cases and less frequent in alcohol-related disorders. Alcohol assessment by AUDIT-C in a hospital setting has a high acceptance rate; in our study, over 95% of eligible patients completed the screening. The findings emphasise the need for universal alcohol screening in hospital settings to detect otherwise hidden cases of AUD, enabling early interventions. A significant proportion of patients with AUD also had mental and behavioural disorders, underscoring the need for integrated care addressing both AUD and mental health. Key factors such as age, sex, emergency admissions, and social determinants were linked to higher odds of AUD, suggesting the need for targeted screening for at-risk groups. The performance of the AUDIT-C score varied among hospitalised patients based on their ICD-10 diagnosis, which should be considered when implementing universal alcohol screening in these settings.

## Figures and Tables

**Table 1:** Baseline characteristics of the cohort.

	No AUD	AUD	*p*-Value
All admissions	54,318 (85.3)	9349 (14.7)	
Individuals	36,350 (83.5)	7164 (16.5)	
Male	16,017 (44.1)	4846 (67.7)	<0.001
Age years (SD)	64.6 (±20.0)	56.0 (±14.2)	<0.001
Ethnicity			<0.001
White	25,826 (89.9)	5168 (94.5)	
Minority ethnicity	2914 (10.1)	300 (5.5)	
Missing	7610	1696	
IMD quintiles			0.195
1 (most deprived)	8797 (26.4)	1759 (26.9)	
2	6094 (18.3)	1169 (17.9)	
3	5749 (17.2)	1056 (16.2)	
4	5475 (16.4)	1094 (16.7)	
5 (least deprived)	7222 (21.7)	1455 (22.3)	
Missing	3013	631	
Civil status			<0.001
In a relationship ^a^	18,108 (60.4)	2783 (48.0)	
Not in a relationship ^b^	11,853 (39.6)	5792 (52.0)	
Missing	6389	1372	
Mode of admission			0.421
Emergency	21,355 (58.7)	4172 (58.2)	
Other ^c^	14,995 (41.3)	2992 (41.8)	
Speciality			<0.001
Medicine	19,942 (55.9)	3335 (47.8)	
Surgery	15,761 (44.1)	3642 (52.2)	
Other or unknown	647	187	
Length of Stay (days)	4 (1–268)	3 (1–178)	<0.001
Number of admissions	2.5 (±3.8)	2.1 (±3.0)	<0.001

Data are *n* (%), mean (SD) or median (range), No alcohol use disorder = AUDIT-C score ≤ 4. Alcohol use disorder = AUDIT-C score ≥ 5. SD—standard deviation, IMD—index of multiple deprivations. ^a^ The ‘in a relationship’ category includes patients who are married, in a civil partnership, or in a long-term relationship. ^b^ The ‘Not in a relationship’ category includes patients who are single, divorced, separated, in a dissolved civil partnership, widowed, or are a surviving civil partner. ^c^ Other modes of admissions were those in which patients did not present directly to the accident or emergency (A&E) department and included elective admissions and admissions from consultants and a GP clinic.

**Table 2:** Wholly attributable alcohol conditions and alcohol use disorder (AUD) ^a^.

Condition	No AUD	AUD	*p*-Value
Wholly attributable alcohol conditions			<0.001
Yes	64 (0.2)	283 (4.0)	
No	36,286 (99.8)	6881 (96.0)	
Mental and behavioural disorders due to use of alcohol (F10.X)	25 (39.1)	183 (64.7)	<0.001
Alcohol intoxication	6 (9.4)	19 (6.7)	
Harmful use of alcohol	1 (1.6)	6 (2.1)	
Alcohol dependence	5 (7.8)	9 (3.2)	
Alcohol withdrawal state	11 (17.2)	137 (48.4)	
Alcohol withdrawal state with delirium	1 (1.6)	10 (3.5)	
Alcohol-induced psychotic disorders	0	0	
Alcohol-induced amnestic disorders	1 (1.6)	2 (0.7)	
Alcohol-induced residual and late-onset psychotic disorders	0	0	
Liver disorders due to alcohol	33 (51.6)	66 (23.3)	<0.001
Alcoholic fatty liver	0	1 (0.4)	
Alcoholic hepatitis	7 (10.9)	23 (8.1)	
Alcoholic fibrosis and sclerosis of liver	0	0	
Alcoholic cirrhosis of liver	16 (25.0)	21 (7.4)	
Alcoholic hepatic failure	9 (14.1)	15 (5.3)	
Alcoholic liver disease, unspecified	1 (1.6)	6 (2.1)	
Gastrointestinal disorders due to alcohol	5 (7.8)	33 (11.7)	<0.001
Alcoholic gastritis	0	7 (2.5)	
Alcohol-induced acute pancreatitis	1 (1.6)	26 (9.2)	
Alcohol-induced chronic pancreatitis	4 (6.3)	0	
Poisoning due to alcohol			
Accidental poisoning by and exposure to alcohol	0	0	
Intentional self-poisoning by and exposure to alcohol	0	0	
Poisoning by and exposure to alcohol; undetermined intent	0	0	
Ethanol poisoning	0	0	
Methanol poisoning	0	0	
Other disorders due to alcohol	1 (1.6)	1 (0.4)	
Alcohol-induced pseudo-Cushing’s syndrome	0	0	
Degeneration of nervous system due to alcohol	0	0	
Alcoholic polyneuropathy	1 (1.6)	1 (0.4)	
Alcoholic myopathy	0	0	
Alcoholic cardiomyopathy	0	0	
Foetal alcohol syndrome (dysmorphic)	0	0	
Excess alcohol blood levels	0	0	
Evidence of alcohol involvement determined by blood alcohol level	0	0	
Evidence of alcohol involvement determined by level of intoxication	0	0	

*n* (%), variables with number of participants < 30 were not analysed. ^a^ As per Public Health England (2014 and 2020) guidance, the conditions were defined as alcohol-specific (wholly alcohol-attributable) where alcohol was the sole cause, and their alcohol-attributable fraction was 1.0 (100 percent).

**Table 3:** Alcohol-related discharge diagnosis and alcohol use disorder (AUD) ^a^.

Condition		Low Risk	AUD	*p*-Value
Alcohol-related	No	29,518 (81.2)	5909 (82.5)	0.011
	Yes	6832 (18.8)	1255 (17.5)	
Infection and parasitic diseases				
	Tuberculosis	16 (0.2)	4 (0.3)	
Malignant neoplasm				0.988
	Lip, oral cavity, and pharynx	124 (1.8)	42 (3.3)	
	Oesophagus	95 (1.4)	18 (1.4)	
	Colon	161 (2.4)	30 (2.4)	
	Rectum	95 (1.4)	22 (1.8)	
	Liver and intrahepatic bile ducts	122 (1.8)	13 (1.0)	
	Larynx	21 (0.3)	5 (0.4)	
	Breast	266 (3.9)	44 (3.5)	
Endocrine				
	Diabetes mellitus (type II)	95 (1.4)	15 (1.2)	
Diseases of the nervous system				
	Epilepsy and status epilepticus	154 (2.3)	13 (1.0)	
Cardiovascular diseases				0.028
	Hypertensive diseases	56 (0.8)	11 (0.9)	
	Ischaemic heart disease	1140 (16.7)	307 (24.5)	
	Cardiac arrhythmias	375 (5.5)	81 (6.5)	
	Heart failure	534 (7.8)	56 (4.5)	
	Haemorrhagic stroke	290 (4.2)	70 (5.6)	
	Ischaemic stroke	691 (10.1)	138 (11.0)	
	Oesophageal varices	13 (0.2)	5 (0.4)	
Respiratory infections				<0.001
	Pneumonia	1816 (26.6)	240 (19.1)	
Digestive diseases				0.388
	Gastro-oesophageal laceration haemorrhage syndrome	5 (0.1)	5 (0.4)	
	Unspecified liver disease	17 (0.2)	5 (0.4)	
	Cholelithiasis (gallstones)	417 (6.1)	64 (5.1)	
	Acute and chronic pancreatitis	217 (3.2)	66 (5.3)	
Skin diseases				
	Psoriasis	6 (0.1)	0	
Pregnancy and childbirth				
	Spontaneous abortion	106 (1.6)	1 (0.1)	

*n* (%), variables with number of participants < 30 were not analysed. ^a^ As per Public Health England (2014 and 2020) guidance, the conditions were defined as alcohol-related (partly alcohol-attributable) when alcohol was contributory but not the sole cause.

**Table 4:** Multivariable logistic regression analysis.

	Univariable	*p*-Value	Multivariable	*p*-Value
Age group (years)				
18–29	1		1	
30–39	0.98 (0.86–1.10)	0.702	1.48 (1.29–1.69)	<0.001
40–49	0.88 (0.79–0.97)	0.011	2.52 (2.19–2.89)	<0.001
50–59	1.29 (1.16–1.43)	<0.001	3.86 (3.32–4.49)	<0.001
60–69	0.99 (0.89–1.10)	0.864	4.19 (3.53–4.99)	<0.001
>70	0.39 (0.36–0.44)	<0.001	2.88 (2.33–3.55)	<0.001
Sex				
Female	0.38 (0.35–0.39)	<0.001	0.34 (0.35–0.39)	<0.001
Male	1		1	
Ethnicity				
Minority ethnicity	0.51 (0.46–0.58)	<0.001	0.39 (0.35–0.45)	<0.001
White	1		1	
IMD quintiles				
1 (most deprived)	0.99 (0.92–1.07)	0.846	0.79 (0.74–0.86)	<0.001
2	0.95 (0.87–1.03)	0.254	0.80 (0.73–0.88)	<0.001
3	0.91 (0.84–0.99)	0.036	0.78 (0.72–0.86)	<0.001
4	0.99 (0.91–1.08)	0.851	0.92 (0.84–1.01)	0.072
5 (least deprived)	1		1	
Civil status				
Not in a relationship ^a^	1.65 (1.56–1.74)	<0.001	1.18 (1.11–1.26)	<0.001
In a relationship ^b^	1		1	
Mode of admission				
Emergency	0.98 (0.93–1.03)	0.421	1.21 (1.14–1.29)	<0.001
Other	1		1	
Speciality				
Medicine	0.72 (0.68–0.76)	<0.001	0.85 (0.80–0.90)	<0.001
Surgery	1			
Length of Stay (days)				
	0.98 (0.97–0.99)	<0.001	0.99 (0.98–1.00)	<0.001

Odds ratio (95% CI), no AUD (low-risk) was set as the reference category. The variables were mutually adjusted within the model. ^a^ The ‘not in a relationship’ category includes patients who are either married, in a civil partnership, or in a long-term relationship. ^b^ The ‘in a relationship’ category includes patients who are single, divorced, separated, in dissolved civil partnership, widowed, or are a surviving civil partner.

## Data Availability

The data presented in this study are available on request from the corresponding author due to privacy, legal and ethical reasons.

## Supplementary Materials

The following supporting information can be downloaded at https://www.mdpi.com/article/10.3390/jcm13247617/s1. Table S1: Alcohol use disorder identification test consumption (AUDIT-C); Table S2: ICD-10 codes for alcohol specific (wholly alcohol-attributable) conditions. Table S3: ICD-10 codes for alcohol related (partly alcohol-attributable) conditions. Table S4: Charateristic of alcohol use disorder (AUD) risk groups. Table S5: Distribution of alcohol specific conditions among different AUD risk groups. Table S6: Adjusted multivariable logistic regression analysis. Figure S1: Top ten inpatient specialty of care: for individual alcohol use disorder (AUD) Subgroups.

## Abbreviations

AUD	Alcohol use disorder.
ARD	Alcohol-related disorders.
ARLD	Alcohol-related liver disease.
CI	Confidence interval.
CQUIN	Commissioning for Quality and Innovation.
SD	Standard deviation.
ICU	Intensive care unit.
GCS	Glasgow coma scale.
NHS	National Health Services.
NICE	The National Institute for Health and Care Excellence.
NCEPOD	The National Confidential Enquiry into Patient Outcome and Death.
ONS	The Office of National Statistics.
PHE	Public Health England.
UK	United Kingdom.
UAS	Universal alcohol screening.
