# Supplementary material for: Screening for Alcohol Use Disorder Among Hospitalised Patients: Learning from a Retrospective Cohort Study in Secondary Care
Source: J Clin Med. 2024 Dec 13;13(24):7617. doi: 10.3390/jcm13247617 (PMC11678479; doi:10.3390/jcm13247617)
Supplement: Supplementary file 1 [file jcm-13-07617-s001.zip › jcm-3362730-supplementary.pdf]

Supplemenatry Materials

Table S1. Alcohol use disorder identification test consumption (AUDIT-C)

Question	Score				
	0	1	2	3	4
How often do you have a drink containing alcohol?	Never	Monthly or less	2 to 4 times per month	2 to 3 times per week	4 or more times per week
How many units of alcohol do you drink on a typical day when you are drinking?	0 to 2	3 to 4	5 to 6	7 to 9	10 or more
How often have you had 6 or more units if female, or 8 or more if male, on a single occasion in the last year?	Never	Less than monthly	Monthly	Weekly	Daily or almost daily
			**Final AUDIT-C score:**		
**Interpretation of AUDIT C score**	**Score**				
Screened positive for alcohol use disorder	≥ 5				
Low risk	0-4				
Increased risk	5-7				
High risk	8-10				
Alcohol dependent	11-12				

Table S2. ICD-10 codes for alcohol specific (wholly alcohol-attributable) conditions

Condition	ICD-10 code
**Mental and behavioural disorders due to use of alcohol (F10.X)**	
Alcohol intoxication	F10.0
Harmful use of alcohol	F10.1
Alcohol dependence	F10.2
Alcohol withdrawal state	F10.3
Alcohol withdrawal state with delirium	F10.4
Alcohol induced psychotic disorders	F10.5
Alcohol induced amnestic disorders	F10.6
Alcohol induced residual and late onset psychotic disorders	F10.7
**Liver disorders due to alcohol (K70.x)**	
Alcoholic fatty liver	K70.0
Alcoholic hepatitis	K70.1
Alcoholic fibrosis and sclerosis of liver	K70.2
Alcoholic cirrhosis of liver	K70.3
Alcoholic hepatic failure	K70.4
Alcoholic liver disease, unspecified	K70.9
**Gastrointestinal disorders due to alcohol**	
Alcoholic gastritis	K29.2
Alcohol-induced acute pancreatitis	K85.2
Alcohol-induced chronic pancreatitis	K86.0
**Poisoning due to alcohol**	
Accidental poisoning by and exposure to alcohol	X45

Condition	ICD-10 code
Intentional self-poisoning by and exposure to alcohol	X65
Poisoning by and exposure to alcohol, undetermined intent	Y15
Ethanol poisoning	T51.0
Methanol poisoning	T51.1
**Other disorders due to alcohol**	
Alcohol-induced pseudo-Cushing's syndrome	E24.4
Degeneration of nervous system due to alcohol	G31.2
Alcoholic polyneuropathy	G62.1
Alcoholic myopathy	G72.1
Alcoholic cardiomyopathy	I42.6
Fetal alcohol syndrome (dysmorphic)	Q86.0
Excess alcohol blood levels	R78.0
Evidence of alcohol involvement determined by blood alcohol level	Y90
Evidence of alcohol involvement determined by level of intoxication	Y91

Table S3. ICD-10 codes for alcohol related (partly alcohol-attributable) conditions

Condition	ICD-10 code
**Infection and parasitic diseases**	
Tuberculosis	A15-A19
**Malignant neoplasm**	
Lip, oral cavity and pharynx	C00-C14
Oesophagus	C15
Colon	C18
Rectum	C20
Liver and intrahepatic bile ducts	C22
Larynx	C32
Breast	C50
**Endocrine**	
Diabetes mellitus (type II)	E11
**Diseases of the nervous system**	
Epilepsy and Status epilepticus	G40-G41
**Cardiovascular diseases**	
Hypertensive diseases	I10-I15
Ischaemic heart disease	I20-I25
Cardiac arrhythmias	I47-I48
Heart failure	I50-I51
Haemorrhagic stroke	I60-I62 I69 (x.0-x.2 only)
Ischaemic stroke	I63-I66 I69 (x.3-x.4 only)
Oesophageal varices	I85
**Respiratory infections**	
Pneumonia	J10-J11 (x.0 only) J12-J15 J18
**Digestive diseases**	
Gastro-oesophageal laceration haemorrhage syndrome	K22.6
Unspecified liver disease	K73-K74
Cholelithiasis (gall stones)	K80
Acute and chronic pancreatitis	K85 K86.1
**Skin diseases**	
Psoriasis	L40 (excl. x.5)
**Pregnancy and childbirth**	
Spontaneous abortion	O03
Low birth weight	P05-P07
**Unintentional injuries**	
Road/pedestrian traffic accidents	V02-V04 (x.1 and x.9 only) V09 (x.2 and x.3 only) V12-V14 (x.3-x.9 only) V19 (x.4-x.6 only) V20-V28 (x.3-x.9 only) V29-V79 (x.4-x.9 only) V80 (x.3-x.5 only) V81-V82 (x.1 only) V83-V86 (x.0-x.3 only) V87 (excl. x.9) V89.2
Poisoning	X40–X49 (excl. X45)
Fall injuries	W00-W19
Fire injuries	X00-X09
Drowning	W65-W74

Condition	ICD-10 code
Other unintentional injuries	V01 V09 (x.0, x.1 and x.9 only) V10-V11 V12-V14 (x.0-x.2 only) V15-V18 V19 (x.1-x.3 only) V20-V28 (x.1 and x.2 only) V29 (x.0-x.3 only) V30-V38 (x.1 and x.2 only) V39 (x.0-x.3 only) V40-V48 (x.1 and x.2 only) V49 (x.0-x.3 only) V50-V48 (x.1 and x.2 only) V59 (x.0-x.3 only) V60-V48 (x.1 and x.2 only) V69 (x.0-x.3 only) V70-V48 (x.1 and x.2 only) V79 (x.0-x.3 only) V80 (excl. x.2-x.5) V81-V82 (excl. x.1) V83-V86 (x.4-x.9 only) V87.9 V88 V89 (excl. x.2) V90-V99 W20-W52 W75-W99 X10 X33 X50-X59 Y40-Y89 (excl. Y87)
Intentional self-harm	X60-X84 (excl. X65) Y87.0
Event of undetermined intent	Y10-Y34 (excl. Y15) Y87.2
Assault	X85-Y09 Y87.1

Table S4. Charateristic of alcohol use disorder (AUD) risk groups

	Low risk	Increased risk	High risk	Dependent	*p*-Value[c]
All admissions	54,318 (85.3)	5497 (8.6)	2466 (3.9)	1386 (2.2)	
Individuals	36,350 (83.5)	4372 (10.0)	1892 (4.3)	900 (2.1)	
Male	16,017 (44.1)	2833 (64.8)	1355 (71.7)	658 (73.1)	<0.001
Age years (SD)	64.6 (± 20.0)	57.1 (± 18.5)	54.5 (± 17.3)	53.8 (± 14.2)	<0.001
Ethnicity					<0.001
White	25,826 (89.9)	3121 (94.8)	1376 (94.4)	671 (93.6)	
BAME	2914 (10.1)	172 (5.2)	82 (5.6)	46 (6.4)	
Missing	7,610	1,079	434	183	
IMD quintiles					<0.001
1 (most deprived)	8797 (26.4)	927 (23.2)	523 (30.2)	309 (38.3)	
2	6094 (18.3)	682 (17.1)	311 (18.0)	176 (21.8)	
3	5749 (17.2)	668 (16.7)	274 (15.8)	114 (14.1)	
4	5475 (16.4)	725 (18.1)	269 (15.6)	100 (12.4)	
5 (least deprived)	7222 (21.7)	995 (24.9)	352 (20.4)	108 (13.4)	
Missing	3,013	375	163	93	
Civil status					<0.001
In a relationship[a]	18,108 (60.4)	1832 (52.3)	695 (35.0)	256 (34.3)	
Not in a relationship[b]	11,853 (39.6)	1669 (47.7)	850 (65.0)	490 (65.7)	
Missing	6,389	871	347	154	
Mode of admission					<0.001
Emergency	21,355 (58.7)	2334 (53.4)	1147 (60.6)	691 (76.8)	
other	14,995 (41.3)	2038 (46.6)	745 (39.4)	209 (23.2)	
Speciality					<0.001
Medicine	19,942 (55.9)	1850 (43.3)	879 (47.8)	606 (69.7)	
Surgery	15,761 (44.1)	2420 (56.7)	959 (52.2)	263 (30.3)	
Other or unknown	647	102	54	31	
Length of Stay (days)	4 (1-268)	3 (1-177)	3 (1- 127)	4 (1-157)	<0.001
Number of admissions	2.5 (± 3.8)	2.1 (± 2.9)	2.1 (± 3.4)	2.2 (± 2.3)	<0.001

Data is *n* (%), mean (SD) or median (range), AUDIT-C score: 0-4 (low risk), 5-7 (increased risk), 8-10 (high risk), 11-12 (alcohol dependent). IMD-index of multiple deprivation

[a]In a relationship includes married, in civil partnership or in long term relationship

[b]Not in a relationship includes single, divorced, separated, dissolved civil partnership, widowed, or surviving civil partner.

[c]p for difference in low risk versus; increased risk, high risk and alcohol dependent

Table S5. Distribution of alcohol specific conditions among different AUD risk groups

Condition		Alcohol use disorder (AUD)			
		Low risk	Increased risk	High risk	Dependent
Wholly attributable alcohol conditions					
	Yes	64 (0.2)	37 (0.8)	68 (3.6)	178 (19.8)
	No	36,286 (99.8)	4335 (99.2)	1824 (96.4)	722 (80.2)
Mental and behavioural disorders due to use of alcohol (F10.X)		25 (39.1)	18 (48.6)	40 (58.8)	125 (70.2)
Liver disorders due to alcohol (K70.x)		33 (51.6)	12 (32.4)	18 (26.5)	36 (20.2)
Gastrointestinal disorders due to alcohol		5 (7.8)	6 (16.2)	10 (14.7)	17 (9.6)
Poisoning due to alcohol		0	0	0	0
Other disorders due to alcohol		1 (1.6)	1 (2.7)	0	0

Table S6. Adjusted multivariable logistic regression analysis

	Increase risk	*p*-Value	High risk	*p*-Value	Dependent	*p*-Value
Age group (years)						
18-29	1		1		1	
30-39	0.97 (0.83-1.15)	0.755	1.24 (0.98-1.56)	0.078	2.35 (1.61-3.42)	<0.001
40-49	1.26 (1.07-1.49)	0.006	1.44 (1.13-1.84)	<0.001	3.43 (2.34-5.02)	<0.001
50-59	1.51 (1.26-1.79)	<0.001	1.95 (1.51-2.52)	<0.001	4.19 (2.88-6.29)	<0.001
60-69	1.59 (1.31-1.95)	<0.001	1.76 (1.31-2.40)	0.003	3.82 (2.41-6.04)	<0.001
>70	1.37 (1.08-1.74)	0.008	1.36 (0.96-1.93)	0.073	3.27 (1.94-5.54)	<0.001
Sex						
Female	0.65 (0.61-0.69)	<0.001	0.57 (0.52-0.63)	<0.001	0.60 (0.53-0.69)	<0.001
Male	1		1		1	
Ethnicity						
BAME	0.68 (0.59-0.78)	<0.001	0.65 (0.53-0.79)	<0.001	0.67 (0.51-0.89)	0.006
White	1		1		1	
IMD quintiles						
1 (most deprived)	0.81 (0.73-0.89)	<0.001	0.97 (0.84 (1.12)	0.724	1.21 (0.98-1.50)	0.073
2	0.84 (0.75-0.93)	0.001	0.93 (0.79-1.08)	0.341	1.16 (0.92-1.46)	0.199
3	0.86 (0.77-0.95)	0.004	0.92 (0.78-1.07)	0.282	1.03 (0.81-1.32)	0.797
4	0.94 (0.85-1.04)	0.279	0.96 (0.82-1.13)	0.660	1.04 (0.81-1.33)	0.739
5 (least deprived)	1		1		1	
Civil status						
Not in a relationship[a]	1.05 (0.97-1.13)	0.256	1.14 (1.02-1.28)	0.025	1.37 (1.16-1.61)	<0.001
In a relationship[b]	1		1		1	
Mode of admission						
Emergency	1.04 (0.96-1.11)	0.330	1.17 (1.06-1.30)	0.003	1.38 (1.17-1.61)	<0.01
Other	1				1	
Speciality						
Medicine	0.85 (0.79-0.91)	<0.001	0.89 (0.79-0.98)	0.027	1.16 (0.99-1.35)	0.066
Surgery	1		1		1	
Length of Stay (days)						
	0.99 (0.98-0.99)	<0.01	1.00 (0.99-1.03)	0.477	1.00 (0.99-1.01)	0.404
Number of admissions						
	0.98 (0.97-0.99)	<0.01	0.98 (0.97-1.00)	0.042	0.98 (0.96-1.01)	0.146

Odds ratio (95% CI), Low risk group was set as reference category

[a] Not in a relationship includes married, in civil partnership or in long term relationship

[b] In a relationship includes single, divorced, separated, dissolved civil partnership, widowed, or surviving civil partner

p for full adjusted multivariable logistic regression analysis

Figure S1. Top ten inpatient specialty of care: for individual alcohol use disorder (AUD) Subgroups

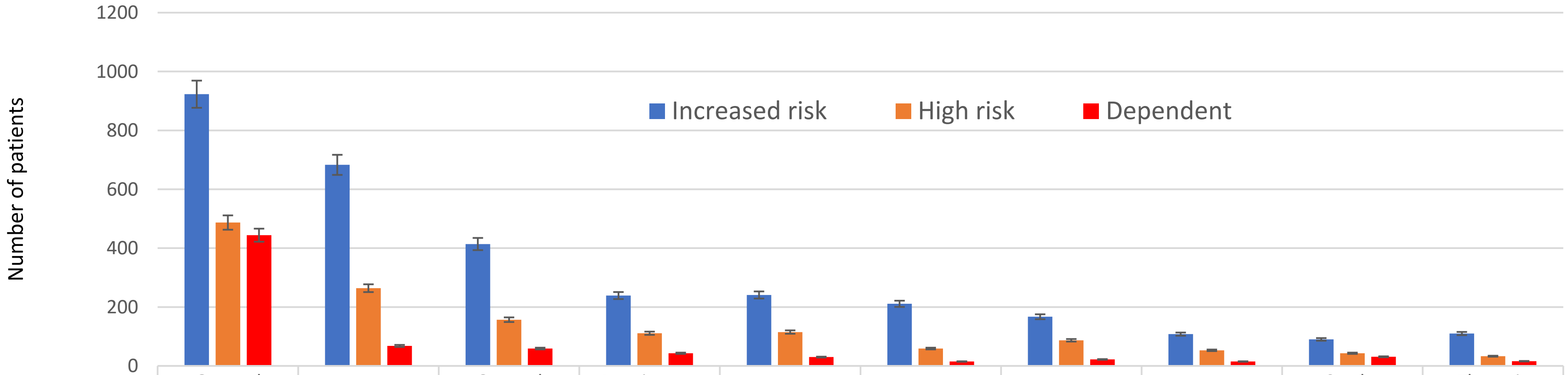

	General Medicine	T&O	General Surgery	Respiratory Medicine	Cardiology	Urology	Neurosurgery	ENT	Stroke Medicine	Thoracic Surgery
Increased risk	923	683	414	239	241	211	167	108	90	110
High risk	487	264	157	111	115	59	87	53	43	33
Dependent	444	68	59	43	30	15	22	15	31	16

Inpatient speciality of care

T&O- Trauma & Orthopaedics, ENT- Ear, nose, and throat (Otorhinology)
